# Rationalization of the Laboratory Diagnosis for Good Management of Malaria: Lessons from Transitional Methods

**DOI:** 10.1155/2022/5883173

**Published:** 2022-04-23

**Authors:** Neguemadji Ngardig Ngaba, Imteyaz A. Khan, Namrata Hange, Maria kezia Lourdes Ligsay Pormento, Manoj Kumar Reddy Somagutta, Ajay Kumar, Youssouf Abdelkerim, Alarangue Djindimadje, Samia Jahan

**Affiliations:** ^1^CHU Bon Samaritain de Walia, N'Djamena, P.O. Box 456, Chad; ^2^Rugers Robert Wood Johnson Medical School, Clinical Academic Building (CAB), 125 Paterson St, New Brunswick, NJ 08901, USA; ^3^Eurasian Cancer Research Council, B - 1210, Golf Scappe, Diamond Garden, Basant Garden, Mumbai, India; ^4^Ateneo de Manila University School of Medicine and Public Health, Don Eugenio Lopez Sr. Medical Complex, Ortigas Ave, Pasig, Metro Manila 1604, Quezon, Philippines; ^5^Avalon University School of Medicine, World Trade Center, Willemstad, Curaçao, Netherlands; ^6^Medstar Union Memorial Hospital, 201 E University Pkwy, Baltimore 21218, Maryland, USA; ^7^Dhaka Medical College, Secretariat Road, Central Shaeed Minar Area, Shahbagh, Dhaka, Bangladesh

## Abstract

**Introduction:**

Malaria is an endemic disease in sub-Saharan Africa. In clinical practice, the main concern is the overdiagnosis of malaria leading to inappropriate drug prescription without laboratory confirmation.

**Objective:**

This study aimed to evaluate clinical examination reliability compared with translational laboratory methods of malaria diagnosis.

**Methods:**

The study was conducted in Goundi Hospital among hospitalized patients over a seven-month period. Patients were interviewed, and malaria tests done included the Giemsa-stained thick and thin blood smears. Diagnostic accuracy was analysed by calculating sensitivity, specificity, and predictive values.

**Results:**

Among 1,874 participants, 674 (35.96%) patients had positive Giemsa-stained thick blood films. The rate of positivity is higher for patients under 5 years of age. The parasite densities were between 160 and 84.000 parasites/*μ*L. The threshold pyrogen of the parasitic density was around 10.000 parasites/*μ*L for patients between 0 and 11 months of age, between 1 and 4 years of age, and between 5 and 14 years of age. This threshold was lower for patients over 15 years of age. The study reported some issues in the findings: 60.88% (607/997) cases of fever without positivity of the blood thick smear and 40.13% (284/674) cases of positivity of the thick drop without fever. The positive predictive value of malaria was between 80 and 85% for patients under 5 years of age. This value is lower for patients between 5 and 14 years of age and patients over 15 years of age.

**Conclusion:**

A presumptive diagnosis of malaria should be confirmed by the laboratory in all suspected cases in all possible scenarios. Every parasitemia should be followed by the calculation of parasitic density. However, for the children under 5 years of age in areas of high transmission, the presumptive diagnosis of malaria in certain circumstances could be considered.

## 1. Introduction

In 2019, there were 229 million cases of malaria globally and 405,000 deaths despite massive efforts for malaria control [1]. The WHO African Region reported a disproportionately high share of the global malaria burden, reflecting 94% [1] of cases and deaths. Malaria is a serious public health threat in Chad with high seasonal malaria transmission with 80% of its population in endemic areas. Chad is one of the African countries paying the heaviest price for malaria with more than 22% of all first-line medical consultations [2]. To avert mortality and ensure prompt management, the practice of presumptive care was widely adopted by many nations. The WHO in its 2006 treatment guidelines recommended the management of malaria based upon clinical suspicion in areas with stable transmission for children less than 5 years [3]. In accordance with the WHO directive, malaria care in some parts of Africa and Asia where the disease burden is the highest was often delivered on the basis of certain symptoms, which were considered malaria symptoms. These symptoms included fever, vomiting, anemia, diarrhea, cough, and polypnea.

The Integrated Management of Childhood Illnesses (IMCI) approach advocates caregivers to combine these symptoms and signs to come up with a relevant approximation regarding the diagnosis of malaria [4]. Unfortunately, these symptoms may be a result of other life-threatening conditions [5] such as meningitis, pneumonia, gastroenteritis, acute viral infection, and pyelonephritis. The hazard of presumptive treatment hence is not just overdiagnosis of malaria, but also confusion and subsequently missed opportunities to correctly treat an ill child. Certain relevant studies [6, 7] demonstrated that patients with symptoms similar to malaria in endemic areas should not always be treated as malaria without laboratory confirmation. Presumptive treatment can indirectly accelerate the emergence of parasite resistance [8] to treatment, resulting in nonmalarial fevers being inappropriately treated with antimalaria drugs, endangering patient life and quality of care. The rational prescription seems urgent. It is therefore necessary to proceed with the use of laboratory tests on subjects presenting probable malaria [9]. WHO recommends that all suspected malaria cases should be confirmed using parasite-based diagnostic testing using either microscopy-Giemsa staining or rapid diagnostic test (RDTs) before administering any treatment [9].

A tough impediment to evidence-based management of malaria is the availability of laboratory confirmation in deprived healthcare settings. However, the launch of malaria RDTs comes up with a solution to this challenge. RDTs are more reliable, easy-to-use, and cheaper diagnostic tests. RDTs have a good sensitivity, require minimal training, and keep their accuracy even after substantial storage under tropical conditions. The performance of RDTs is comparable to that of expert microscopy according to relevant studies [10, 11].

The health system in Chad is a pyramid system with three [12] levels of responsibility and activities and places particular emphasis on the Health District: the central level, which includes the National Health Council and a central administration; the intermediate level, which includes regional health councils and regional health delegations; the peripheral level, which includes the District Hospital Councils and district hospitals.

The role of the central level is to design and guide health policies. It is responsible for mobilizing and coordinating national and international resources, supervising, evaluating, and controlling the implementation of national and intermediate level programs. It is made up of referral hospitals and training centers.

The intermediate level is responsible for coordinating the implementation of the national health policy and provides technical support at the peripheral level. It is made up of regional hospitals and training centers.

The peripheral level is responsible for the execution of the activities of the Complementary Package of Activities (district hospitals) and the Minimum Package of Activities (health centers) and constitutes the operational level of the national health policy.

A meaningful study [2] performed by Othingué et al. in Chad reported that clinical diagnoses of malaria detect only 30% of malaria cases in patients.

In an attempt to provide a contribution to this debate in our context, this study conducted in Chad aims to rationalize the diagnosis for a good management of malaria for patients at Goundi Hospital. For this reason, the study determines the prevalence of malaria in patients hospitalized at the hospital, then establishes a correlation between the level of parasitemia and the clinical symptoms of the malaria crisis, and confirms the cases of presumptive malaria by parasitological examination.

## 2. Material and Methods

### 2.1. Study Design, Settings, and Timeframe

This study was conducted over a duration of seven months from June 1, 2011, to December 31, 2011, in Goundi Hospital. There is a high transmission of malaria between September and December, hence more malaria cases that would concurrently lead to high misdiagnosis.

The Goundi district hospital consists of a district hospital [12] with a capacity of 120 beds and 9 health centers (of which 3 cover the area of our survey). The management of this district has been entrusted to a local association, the Chadian Community Association for Progress (ATCP). It covers the administrative subprefectures of Goundi and Ngangara. It is attached to the regional health delegation of Koumra. Each health center has an area of responsibility, and each village is therefore affiliated with a health center. The activities in the health centers are, among other things, curative consultation, antenatal consultation (ANC), and vaccination sessions. Routine long-lasting insecticide-treated net (LLIN) distribution and malaria information occur during ANC and immunization sessions.

Our study was carried out at the Goundi Hospital. Goundi Hospital is a district hospital, located in the south of the country, in the Mandoul Oriental Region, 657 km from the Chadian capital (N'Djamena). The statutory policies that govern it assign three objectives: to provide curative care, to provide preventive care, and to promote the training of health workers.

### 2.2. Study Population

Research participants were hospitalized patients of all ages and genders from all four departments of Goundi Hospital including the intensive care unit, the post-intensive care unit, the infectious disease unit, and the gyneco-obstetric unit.

### 2.3. Inclusion Criteria

Hospitalized patients who had not received any antimalarial treatment in the two weeks prior to hospital admission, and who had consented to participate in this study, were included. Only the first admission of a patient was included in the study.

### 2.4. Exclusion Criteria

We have excluded patients who were not hospitalized during the period of the study. Patients who had received antimalarial treatment from another healthcare facility or by automedication during the two weeks prior to their hospitalization, during the study period, were excluded. We have excluded patients who had not consented to participate in the study. Second and subsequent admissions of the same patient were also excluded.

### 2.5. Ethical Considerations and Informed Consent

The study was conducted in accordance with the Declaration of Helsinki (1964). Hospital management and all clinical department heads in hospitals were briefed about the study protocol, data collection tools, and details of planned blood investigations. The study was conducted after authoritative ethical approval from a local institutional research body—hospital committee, including senior management and clinical departmental heads. The research participants were informed about the purpose of the study, along with the planned laboratory tests. Informed oral consent was taken after a briefing about the purpose and protocol of the study. Privacy and confidentiality regarding information were assured to patients and or family. Only the primary medical team, involved in the patient's direct care, was given access to patients' blood results for the continuation of medical care. Once patients had consented to the data collection, the laboratory investigation, and the disclosure of data to the primary treating team, they were allowed to participate in the study. In the case of children, parents were briefed as above. The parent's consent was obtained, and information was collected from parents and/or the children's families. In the case of seriously ill or incapacitated patients, consent was taken and data were retrieved from their next of kin.

### 2.6. Data Collection

Patients and/or their next of kin were updated about planned laboratory tests. Basic information was collected through well-designed data collection sheets through direct interviews with the patients or their next of kin. The data collection sheet included information regarding age, gender, fever, referral, health center, and its distance. Laboratory results included thick blood smear/drop and parasite density.

### 2.7. Laboratory Method Technique and Measurements

#### 2.7.1. Temperature Measurement

We used a thermometer to measure the temperature: a rectal temperature threshold of 38.0°C, which was used as a cutoff point for fever [13, 14] in children, but the threshold temperature was 0.5°C lower than rectal when measuring the axillary temperature [15]. For adults, the axillary temperature measured was considered to be a fever when it was more than 37.0°C [16].

#### 2.7.2. Laboratory Procedure Staining of Thick and Thin Smears

Two blood slides were used for each sample that arrived at the laboratory. Each of the slides had a 6 *μ*l volume of blood for thick film and 2 *μ*l for the thin film. Once the thin smear is fixed with methanol, the thin and thick blood smears were stained with Giemsa.

The 10% Giemsa stain was used to stain for 15 minutes on one of the two slides for the first slide reading, and the results were sent to physicians for patients' management. The second slide stained with 3% Giemsa for 60 minutes was given to two independent malaria microscopists. A positive smear was associated with each new cluster of working Giemsa stains, for quality control.

### 2.8. Examination of Thick/Thin Smears

The Giemsa-stained smear was first screened at a low magnification (10X × 40X objective lens) to detect suitable fields with even distribution of white blood cells (WBCs) (10–20 WBC/field) [17, 18]. Complete blood counts, particularly WBC counts, were performed manually using stains, the microscope, and the Neubauer chamber and counters [19, 20].

Smears were afterward examined using X100 oil immersion. At least 100 high power fields were examined before a thick smear was confirmed negative. *Plasmodium falciparum* parasites were counted per 200 or 500 leukocytes; this was done to estimate the parasite density per microliter of blood.

### 2.9. Estimation of Parasite Density

Positive slides were subjected to the parasite density calculation. Parasite densities were reported as a ratio of parasites to WBC in thick films; this gave us the number of parasites per microliter of blood. The number of *Plasmodium* parasites was counted against 200 WBC on the thick film. Parasite densities (parasite/*μ*l of total blood) were then calculated as follows: (number of parasites counted/WBC counted) × WBC count/*μ*L of patient [21].

### 2.10. Outcome Variable

This study's primary interest is the diagnostic accuracy of malaria tests with positive thick and thin smear results and parasite density for malaria. A secondary interest includes the significance of testing parasite density.

### 2.11. Independent Variable

The independent variables include demographic factors such as age group and gender. The clinical profile includes the reporting of fever symptoms and the distance of referral health centers from the study site.

### 2.12. Data Analysis

Data were manually entered into the Microsoft Excel 10 system. Exhaustive data quality control was carried out by the reviewers to ensure the data's correctness and completeness before analysis. The results are expressed in terms of number and percentage. Sensitivity, specificity, positive predictive value (PPV), negative predictive value (NPV), and likelihood ratio were calculated for both thick smears with Giemsa stain and checked for their association with independent sociodemographic and clinical variables. True positives (TP/*a*) are defined as the number of febrile patients with a positive thick drop. True negatives (TN/*d*) are the number of afebrile patients with a negative thick drop. False positives (FP/*c*) are febrile patients who have a negative thick drop, and false negatives (FN/*b*) are patients who are afebrile with a positive thick drop. Sensitivity was calculated as TP/(TP + FN). Specificity was calculated as TN/(TN + FP). The positive predictive value (PPV) was calculated as TP/(TP + FP) and the negative predictive value (NPV) as TN/(FN + TN). McNemar's test was used for further analysis to compare the sensitivities and specificities for evaluating diagnostic tests and presumptive malaria.

We calculated the quartiles (*Q*1, *Q*2, and *Q*3) of parasite density to estimate the scattering of the parasite densities for each age group [22]. The quartiles (*Q*1, *Q*3, and *Q*3) are the parasite densities that divide the ordered parasite densities observed in our sample into four equal groups.

The first quartile (*Q*1) is defined as the middle number between the smallest number (minimum) and the median of the data set. It is also known as the lower or 25th empirical quartile; that is, 25% of the data are below this point. The second quartile (*Q*2) is the median of a data set; thus, 50% of the data lie below this point. The third quartile (*Q*3) is the middle value between the median and the highest value (maximum) of the data set. It is known as the upper or 75th empirical quartile; that is, 75% of the data lie below this point.

## 3. Results

### 3.1. Patient Recruitment and Their Demographic Profile of Study Participants

Participants were predominantly female (53.74%, 1007/1874) (Table 1). The most representative age group is that aged 15 years and above (49.10%, 920/1874) (Table 1).

#### 3.1.1. Healthcare Access

A total of 1618 (86.34%) (Table 2) patients belonged to the Goundi Hospital coverage area. This coverage was composed of the primary healthcare facilities that are located between 1 and 32 km from the hospital. Goundi Hospital had a referral flow of patients from other small towns, which was represented by 256 patients (13.66%) (Table 2).

#### 3.1.2. Laboratory Findings Related to Fever Profile of the Patients

Table 1 shows that the positivity of thick drop was predominant in patients aged between 1 and 4 years, with 46.57% (204/438) of cases. They were followed by 42.21% (141/334) (Table 1) of patients between 0 and 11 months of age. The age groups between 0 and 11 months of age and between 1 and 4 years of age made up more than 50% of the total malaria-positive thick drop.

The combination of fever and positive thick drop was predominant in children as follows: between 0 and 11 months of age and between 1 and 4 years of age, with, respectively, 85.10% (120/141) (Table 3) and 80.39% (164/204) (Table 3) of cases. The other age groups had the highest rate of positive thick drop without fever: there were 74.91% (200/267) (Table 3) of cases for patients between 15 years of age and older and 37.10% (23/62) (Table 3) for patients between 5 and 14 years of age. Age-specific indicators for diagnostic efficacy and accuracy are explained in Table 3.

The McNemar test was used to analyze two variables: the thick peripheral blood smear and the presumptive diagnosis based on fever. Since the *p* value of the McNemar test was <0.000001, we rejected the null hypothesis that the difference in results is not significant. There was a significant difference between the positivity rate and the negativity rate between the two tests. Hence, there is a significant difference between having a peripheral blood smear-positive and presumptive malaria diagnosis. The McNemar test calculated 116.37 with a *p* value of <0.000001.

### 3.2. Parasite Density of the Study Population

The study showed that of the 674 patients testing positive on thick smear, there were 235 (34.86%) (Table 4) cases with a parasite density of more than 10,000 parasites/*µ*L. The remaining 439 (65.13%) (Table 4) patients had a parasite density of less than 10,000 parasites/*μ*L.

#### 3.2.1. Analysis of Parasite Density

*(1)*. *Position Parameters (Median) and Dispersion Parameters (Quartiles)*. The position of the median was within a threshold of significant parasitemia (>10,000P/*μ*L) (Table 5) for the following patients: between 0 and 11 months of age and those between 1 and 4 years of age. This was consistent with the published studies, which identify children under 5 years of age as those most at risk of severe malaria.

### 3.3. Dispersion of Parasite Densities in the Study Population

**Figure 1:**
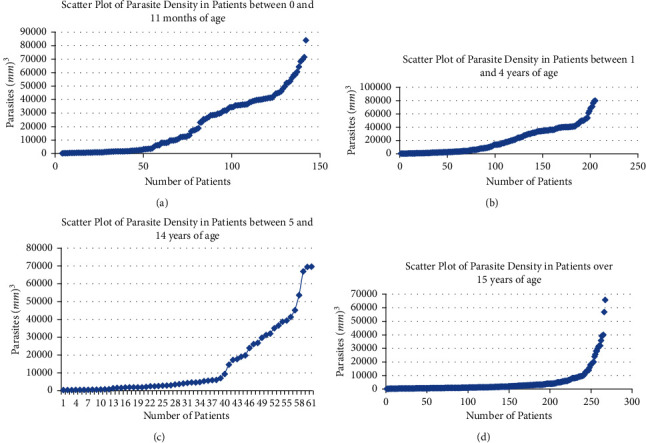
**:** Scatter plot of parasite density for the study population.

There were about 1/3 of cases present with very low parasitemia at 0–11 months (Figure 1). The transition was rapid, and the curve took a breakneck ascending pace, while two thirds of patients in this age group of 0–11 months have significant parasitemia and therefore potentially severe malaria.

The parasitic dispersion for the age group between 1 and 4 (Figure 1) showed one fourth of the patients with low parasitemia. For one fourth of patients, the study reported that the transition to high parasitemia occurred reasonably quickly; the remaining half of the population had significant parasitemia.

The graph for ages between 5 and 14 (Figure 1) showed that two thirds of patients had a parasite density of less than 10,000 parasites/mm^3^. It was the remaining one third who had parasitemia that had entered the clinical threshold for severe malaria. Simultaneously, three fourths of patients over 15 years of age (Figure 1) had a parasite density of less than 10,000 parasites/*μ*L. For the remaining patients that are over 15 years of age, only one-fourth had significant parasitemia.

### 3.4. Study of Parasite Density Expressed as a Percentage of Parasitized Red Blood Cells

**Figure 2:**
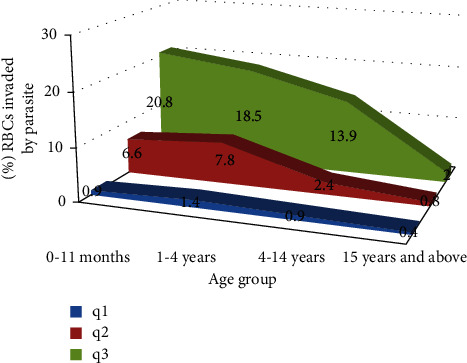
Distribution of parasite density in the percentage of red blood cells.

The study found that in patients under 5 years of age, the median of the percentage of parasitized red blood cells was greater than 5% (Figure 2), considered as a sign of severe malaria.

## 4. Discussion

This study was conducted at Goundi, in an area with a very well-organized healthcare system implemented many years ago. The Goundi district hospital is a health system with a good reputation for the healthcare workers and the involvement of the whole community of the area. The electricity supply is possible at certain times of the day, obliging the laboratory of the hospital to just work during this period of time.

Our results are similar to those of Asseda et al. [23] in northeastern Ethiopia that reported that 53% (95% CI: 50.4–54.2) of the samples tested were from females. Our study found that children under 5 years of age represented 41.19% (772/1874) of the population. This is consistent with significant studies [24, 25] that identified children under five years as those most at risk for severe malaria.

The vast majority of the study cohort comprised of patients from Goundi Hospital itself (86.34%), while the remaining 256 (13.66%) patients came from other towns. This is consistent with the latest statistics from the hospital, which has reported the number of out-of-district hospitalized patients fluctuating between 11 and 13% for the last decade. Patient's trust and healthcare access to Goundi Hospital for inpatient care were justified possibly because of the quality of health service delivery with trained and experienced nursing and medical staff; the availability of inpatient and emergency services and diagnostic services; and subsidized services for the patients.

This study reported that 53.20% of the study participants (997 of 1874 patients) had fever. Based on this result, and just by performing a thick drop without even the parasite densities count, the presumptive diagnosis of malaria taking fever as the main argument would be a serious malpractice without any laboratory test for confirmation. According to the study of Bisoffi Z [26], not all of their 41/127 febrile parasitemia patients are due to malaria. In our study, an error of 60.89% will be expected if we maintain the premise: any fever equals presumptive malaria. Our results were lower than those reported by Manguin S. et al. [27] in 2017 in Angola that reported an average rate of error of 85%. Significant studies [28] recommended the parasitological confirmation of suspected malaria before starting treatment.

This study reported 35.96% (674) of patients with a positive thick blood smear. Our study result was lower than that found by Tuasha N. et al. in 2019 [29] in Ethiopia (64.63%, 276/427). Of the 674 positive thick smears in this study, 390 (57.86%) were associated with fever. Among the febrile cases with positive thick smears, 72.82% were children under five years of age. The PPV of fever as an indicator of malaria was 85% for the study population between 0 and 11 months of age and 80% for children between 1 and 4 years of age. Related studies [30] found that in children, a clinical diagnosis based on fever had a PPV of 2% compared with laboratory results during the dry season, but the PPV rose to 44% during the rainy season. Our study reported a PPV of 62.90% and 25.09%, respectively, for patients between 5 and 14 years of age and patients over 15 years of age.

Our results argue in favour of the principle that the presumptive diagnosis of malaria in areas with high malaria transmission can only be applied to children between 0 and 4 years of age and during the rainy season [30]. For adolescents and adults, attempting to diagnose based on fever too often will result in a false positive, leading to overuse of drugs and subjecting patients to resistance pressure for the antimalarial treatments used [31].

The parasite density was too fluctuating in our study. The parasite density varied from 160 to 84,000 parasites/mm^3^, which made an analysis/interpretation complex. The literature [32] sets a pyrogenic threshold of malaria at around 10.000 parasites per mm3, corresponding to a percentage of parasitized red blood cells of 5%. Our study showed that the age groups most often exposed to severe malaria (under 5 years of age) had a median above this threshold. The age group of 5–14 years had a median below, and this parameter was very low for adults (15 years and over). Fever with malaria rarely appeared below this threshold. The “pyrogenic” threshold was reported in 235 (34.86%) study participants with a positive thick peripheral blood smear, of which 78.71% were children under 5 years of age, including 47.23% for patients between 1 and 4 years of age and 31.48% for patients between 0 and 11 months of age. Our observation between the pyrogenic threshold correlation and parasite density was similar to the study of Smith T. et al. [33] in 2006 that found in their model the occurrence of clinical attack of malaria related to the peripheral parasite densities through a pyrogenic threshold that correlated with the parasite load. However, it is essential to point out that this threshold, which varies according to the epidemic facies, allows the definition of malaria access, but should not be considered an absolute criterion in all cases.

## 5. Conclusion

The presumptive malaria diagnosis will be of immense value only if confirmed by laboratory examination. The postulate of fever as a diagnosis of malaria is far from the truth. The positive predictive value of fever as an indicator of malaria is 80 to 85% for children under 5 years old. After 5 years, it falls to lower levels. For the other age groups, a whole clinical set is needed and, if possible, a biological examination corroborates the diagnosis. This would prevent overprescription of antimalarials and reduce the rising resistance to drugs created by indiscriminate and unjustified use of antimalarial drugs. Considering the cost of rapid diagnostic tests, the test that appears to be the most cost-effective from an economic standpoint is a microscopic diagnostic test. An effort must be made by the healthcare workers to redefine this examination properly. Failure to quantify parasite density makes interpretation of the test difficult. The interpreted result without quantifying the parasite density exposes a risk of overtreatment. We noticed in this study a large number of patients who had a basal parasitemia (65.13%). Weak parasitemia may be accompanied by low gametocyte load that fuels transmission; thus, it is critical to treat infection regardless of parasitemia. The behavior encountered in practice among caregivers is a positive microscopic malaria test equals the prescription of an antimalarial. This habit of prescribing does not correlate with medicine-based evidence practice.

## Figures and Tables

**Table 1:** Gender, age, and malaria test outcomes of the study population.

Gender and age of the study population					

Gender/age	0–11 months	1–4 years	5–15 years	>15 years	Total
Male	183	244	108	332	867 (46.26%)
Female	151	194	74	588	1007 (53.74%)
Total	334 (17.82%)	438 (23.37%)	182 (9.71%)	920 (49.10%)	1874 (100%)
Results of blood smear malaria test of the study population			
Age	Size	Positive malaria test	% of +** **malaria test
0–11 months	334	141	42.21%
1–4 years	438	204	46.57%
5–15 years	182	62	34.06%
Over 15 years	920	267	29.02%
Total	1874	674	35.96%
Predictive value of fever for malaria in the study population			
Fever	Malaria positive	Malaria negative	Total
Present	390 (a)	607 (b)	997 (*a *** **+** ***b*)
Absent	284 (c)	593 (d)	877 (*c* + d)
Total	**674 (a + c)**	**1200 b** **+** **d)**	**1874 (a+b** **+** **c** **+** **d)**
Sensibility: a/(*a*+*b*) 390/997 = 0.39 (39%)			
Specificity: d/(*c* + d) 593/877 = 0.57 (67.61%)			
Positive predictive value (PPV): a/(*a*+c) = 390/674 = 0.57 (57.86%)			
Negative predictive value (NPP): d/(*b* + d) = 593/1200 = 0.49 (49.41%)			
Parasite densities of the study population			
Parasite density/age	Above 15 years	5–14 years	1–4 years	0–11 months	Total
>10.000 parasites/*µ*L	28	22	111	74	**235**
<10.000 parasites/*µ*L	240	40	93	66	**439**

TP: a, FN: *b,* FP: *c,* TN: d.

**Table 2:** Facilities' origin of the study population.

Name of healthcare facilities	Number of patients	%	Distance in kilometer
Goundi-Ouest	422	22.5	1
Goundi-Est	332	17.71	1
Goundi-Nord	237	12.64	3
Mahimtoky	171	9.12	23
Ngangara	140	7.47	18
Guiditi	121	6.45	32
Koumaye	103	5.49	35
Goundi-Sud	92	4.9	9
Referral from other towns	256	13.66	>35

**Table 3:** Laboratory findings related to fever profile of the patients.

Distribution by age group of patients with positive malaria according to fever			

Age	Positive malaria with fever (%)	Positive malaria without fever (%)	Total size (%)
0–11 months	120 (85.10)	21 (14.89)	141 (100)
1–4 years	164 (80.40)	40 (19.60)	204 (100)
5–14 years	39 (62.90)	23 (37.10)	62 (100)
>15 years	67 (25.09)	200 (74.91)	267 (100)
Total	390	284	674 (100)
Predictive value of fever for malaria in patients between 0 and 11 months of age			
Fever	Positive malaria	Negative malaria	Total
Present	120 (a)	158 (b)	278 (a + b)
Total	141 (a + c)	213 (b + d)	334 (a + b + c + d)
Sensitivity: a/(a + b) 120/278 = 0.43 (43%)			
Specificity: d/(c + d) 55/76 = 0.72 (72%)			
Positive predictive value: a/(a + c) = 120/141 = 0.85 (85%)			
Negative predictive value: d/(b + d) = 55/213 = 0.25 (25.82%)			
Predictive value of fever for malaria in patients between 1 and 4 years of age			
Fever	Positive malaria	Negative malaria	Total
Present	164 (a)	161 (b)	325 (a+b)
Absent	40 (c)	73 (d)	113 (c + d)
Total	204 (a + c)	234 (b + d)	438 (a +b + c + d)
Sensitivity = 164/325 = 0.50 (50.46%)			
Specificity = 73/113 = 0.64 (64.60%)			
Positive predictive value = 164/204 = 0.80 (80.39%)			
Negative predictive value = 73/234 = 0.31 (31%)			
Predictive value of fever for malaria in patients between 5 and 15 years of age			
Fever	Positive malaria	Negative malaria	Total
Present	39 (a)	63 (b)	102 (a + b)
Absent	23 (c)	57 (d)	80 (c + d)
Total	62 (a + c)	120 (b + d)	182 (a + b + c + d)
Sensitivity = 39/102 = 0.38 (38%)			
Specificity = 57/80 = 0.71 (71%)			
Positive predictive value = 39/62 = 0.63 (63%)			
Negative predictive value = 57/120 = 0.47 (47.5%)			

**Table 4:** Parasite density according to age group.

Age group	Parasite density >10,000	%	Parasite density <10,000	%
0–11 months	74	31.49	66	15.03
1–4 years	111	47.23	93	21.18
5–14 years	22	9.61	40	9.112
>15 years	28	11.91	240	54.67
	235	100	439	100

**Table 5:** Dispersion around the mean of the parasite densities for each age class.

Age/parasite density	Q1	Q2	Q3
0–11 months	1640	12240	36320
1–4 years of age	2460	13800	35060
4–14 years of age	1680	4400	25050
15 years and more	800	1520	3520

## Data Availability

The authors confirm that all data underlying the findings are fully available without restriction. All relevant data are within the manuscript.
